# The Fto Gene Regulates the Proliferation and Differentiation of Pre-Adipocytes *in Vitro*

**DOI:** 10.3390/nu8020102

**Published:** 2016-02-19

**Authors:** Yang Jiao, Jingying Zhang, Lunjie Lu, Jiaying Xu, Liqiang Qin

**Affiliations:** 1 Collaborative Innovation Center of Radiological Medicine of Jiangsu Higher Education Institutions, Medical College of Soochow University, Suzhou 215123, China; jiaoyang@suda.edu.cn (Y.J.); xujiaying@suda.edu.cn (J.X.); 2 Department of Radiation Genetics, School of Radiation Medicine and Protection, Medical College of Soochow University, Suzhou 215123, China; 15850122119@163.com (J.Z.); llj19900622@163.com (L.L.); 3 Department of Nutrition and Food Hygiene, School of Public Health, Medical College of Soochow University, Suzhou 215123, China

**Keywords:** obesity, Fto, pre-adipocyte, proliferation, differentiation

## Abstract

The highly regulated differentiation and proliferation of pre-adipocytes play a key role in the initiation of obesity. Fat mass and obesity associated (*FTO*) is a novel gene strongly associated with the risk of obesity. A deficiency of FTO may cause growth retardation in addition to fat mass and adipocyte size reduction *in vivo*. To investigate the potential role of *Fto* gene on the proliferation and differentiation of pre-adipocytes, we generated Fto-knockdown and overexpressed 3T3-L1 cells. Using numerous proliferation assays our results suggest that Fto knockdown leads to suppression of proliferation, lower mitochondrial membrane potential, less cellular ATP, and decreased and smaller intracellular lipid droplets compared with controls (*p* < 0.05). Western blot analysis demonstrated that Fto knockdown can significantly suppress peroxisome proliferator-activated receptor gamma (PPARγ) and glucose transporter type 4 (GLUT4) expression and inhibit Akt phosphorylation. By contrast, overexpression of *Fto* had the opposing effect on proliferation, mitochondrial membrane potential, ATP generation, *in vitro* differentiation, Akt phosphorylation, and PPARγ and GLUT4 expression. Moreover, we demonstrated that Wortmannin, a phosphoinositide 3-kinase (PI3K) inhibitor, could inhibit phospho-Akt in Fto overexpressed 3T3-L1 cells. Taken together, the results suggest that Fto regulates the proliferation and differentiation of 3T3-L1 cells via multiple mechanisms, including PPARγ and PI3K/Akt signaling.

## 1. Introduction

Obesity increasingly is a pressing public health concern with a rapidly surging prevalence in both developed and developing countries [1,2,3]. According to the World Health Organization, in 2014 more than 1.9 billion adults were overweight, 600 million of whom were obese [4]. In addition to psychosocial ramifications of obesity, this condition causes a multitude of health complications [1,2,3]. It has been widely reported that the majority of obese individuals have multiple comorbidities including inflammation, hypertension, coronary heart disease and stroke, type 2 diabetes mellitus, insulin resistance, gall bladder disease, nonalcoholic fatty liver disease, and cancers, among others. Together, these result in the poor health of those affected and may result in accelerated aging and death [3,5].

Unfortunately, the existing interventions for obesity control, including diet modification and exercise-based managements, seem largely ineffective [5], in part related to our inadequate and incomplete understanding of mechanisms of pathogenesis. It has been well accepted that adipose tissues play an integral role in lipid and glucose metabolism; therefore precise regulation of differentiation and proliferation of pre-adipocytes are thought to play a key role in the initiation of obesity [6,7,8,9,10,11]. Recently, the molecular mechanisms and signaling pathways regulating pre-adipocytes differentiation and proliferation have been extensively studied; however, our understanding of these processes remains incomplete [10,12].

The fat mass and obesity associated gene (*Fto*) is an important genes that is strongly associated with the risk for obesity [13,14]. *In vivo* experiments have demonstrated that *Fto*-deficiency causes growth retardation in addition to fat mass and adipocyte size reduction [15]. Furthermore, overexpression or dysregulation of *Fto* alleles is associated with increased risk of obesity while inhibition of *Fto* may have a protective-effect against obesity in animal models [15,16,17]. Recently, several studies have highlighted the importance of *Fto* gene during adipogenesis [18,19,20]. Zhao *et al.* [18] suggested *Fto* can modulate *SRSF2*-RNA binding and demethylation of N6-methyladenosine, which together impact RNA splicing and critically regulate adipogenesis. Tews *et al.* [19] hypothesized that *Fto* deficiency may lead to the induction of white adipose tissue (WAT) browning and in turn cause mitochondrial uncoupling and increase energy expenditure. Taken together, this suggests an FTO-associated susceptibility to obesity. Merkestein *et al.* [20] reported that FTO promotes adipogenesis via mitotic clonal expansion. However, the specific mechanisms and signaling pathways by which FTO impacts adipogenesis and obesity remains incompletely characterized. 

In the present study, the pre-adipose cell line 3T3-L1 was utilized as an *in vitro* model, and the biological roles and potential mechanisms of Fto on the proliferation and differentiation of pre-adipocytes were evaluated via classical gain/loss-of-function experiments.

## 2. Materials and Methods

### 2.1. Cell Culture and Transfection 

The 3T3-L1 cell line was originally obtained from American Type Culture Collection (ATCC, Manassas, VA, USA). Cells were cultured in high-glucose Dulbecco’s modified eagle’s media (Gibco, Grand Island, NY, USA), supplemented with 10% fetal bovine serum, L-glutamine (2 mmol/L), non-essential amino acids (2 mmol/L), penicillin (100 U/mL) and streptomycin (100 U/mL) (Gibco, Grand Island, NY, USA). Cells were grown in a humidified atmosphere with 5% CO_2_ at 37 °C. Culture medium was replaced every 2 days, and cells were sub-cultured every 3–4 days. Used for this study are 3T3-L1 cells within 15 passages.

Using the genomic sequence of the mouse *Fto* gene (GeneBank ID: 26383), an *Fto* recombinant plasmid construction was constructed as previously described [21]. Three Fto siRNAs and a negative control siRNA (NC siRNA) were designed and synthesized (Invitrogen, Carlsbad, CA, USA). The Fto inhibition efficiency by siRNA was evaluated by qRT-PCR and Western blot assay. The siRNA #2 elicited the most effective suppression of Fto and was selected for subsequent studies. The sequences for Fto-siRNA #2 is as follows (5’ to 3’):

GCAUGUCAGACCUUCCUAATT
UUAGGAAGGUCUGACAUGCTT.

The siRNA and plasmid transfections were performed using Lipofectamine® 2000 (Thermofisher scientific, Waltham, MA, USA) according to the manufacturers’ instructions. Briefly, 3T3-L1 cells (2 × 10^5^) were seeded onto 6-well plates and incubated overnight. Then 30nM siRNAs and NC control, as well as 0.5 μg plasmid DNA were applied to each well together with recommended volume of transfection reagent. Cells were harvested at indicated time points following transfection. 

### 2.2. Reverse Transcription and Quantitative Real Time PCR 

Total RNA was isolated according to the TRIZOL method (Invitrogen, Carlsbad, CA, USA). First-strand cDNA was synthesized from 1 μg of total RNA using Hiscript^TM^ 1st strand cDNA synthesis kit (Vazyme, Nanjing, CHN). Real time PCR was performed in Applied Biosystems 7500FAST RT-PCR system using SYBR Green (Thermofisher scientific, Waltham, MA, USA). The sequence of all Primers was as follows: *Fto*-Forward, 5’-GACACTTGGCTTCCTTACCAG-3’ and *Fto*-Reverse, 5’-CTCACCACGTCCCGAAACAA-3’; and *GAPDH*-Forward, 5’-GGTTGTCTCCTGCGACTTCA-3’, and *GAPDH*-Reverse, 5’-TGGTCCAGGGTTTCTTACTCC-3’. The expression of the *Fto* gene was calculated with the method of 2^−∆∆CT^.

### 2.3. Western Blot Assay

As previously described [22], cells were collected, washed in ice-cold PBS and lysed in 50 μL of lysis buffer with protease inhibitor cocktail (Roche Life Science, Indianapolis, IN, USA). The protein concentration was determined using the BSA assay, and 50 μg total protein for each sample was separated by SDS-polyacrylamide gel electrophoresis. The protein was transferred onto polyvinylidene fluoride (PVDF, Millipore, Billerica, MA, USA) microporous membranes, incubated with primary antibody overnight at 4 °C, and subsequently incubated with the corresponding secondary antibodies. The primary antibodies used include rabbit anti-FTO (5-2H10, Abcam, Cambridge, MA, USA, 1:1000), rabbit anti-PPARγ (Abcam, polyclonal, ab19481, 1:400), rabbit anti-phospho-Akt (Ser473) (D9E, cell signaling technology, Danvers, MA, USA, 1:2000), rabbit anti-Glucose Transporter GLUT4 (Abcam, polyclonal, ab654, 1:2000), mouse anti-AMPK (Abcam, 34.2, 1:1000), rabbit anti-Adipose Triglyceride Lipase antibody (ATGL) (Abcam, EPR3444(2), 1:1000), HSL Antibody (cell signaling technology, polyclonal, 4107, 1:1000), rabbit anti-CREB (cell signaling technology, 48H2, 1:1000), rabbit anti-phospho-CREB(Ser133) (cell signaling technology, 87G3, 1:1000) and anti-β-actin (Beyotime Biotechnology, Haimen, China). Secondary antibodies include goat anti-mouse and anti-rabbit HRP (horseradish peroxidase)-conjugated antibodies (1:1000, Beyotime Biotechnology, Haimen, China). 

### 2.4. Cell Viability Assay 

As previously described, MTT (3-(4,5-Dimethylthiazol-2-yl)-2,5-diphenyltetrazolium bromide, a tetrazole) was used to evaluate cell viability [22]. In brief, cells were seeded at a density of 5 × 10^4^ cells/well in 24-well plates. Following various treatments, 5 mg/mL MTT (Sigma-Aldrich, MO, USA) was added at indicated time points. After 4 h of incubation, the supernatant was discarded, and DMSO was added to dissolve the formazan generated. Absorbance at 490 nm was measured using multimode microplate reader (BioTek, VT, USA). Cell viability was expressed as the percentage of viable cells with different treatments relative to cell viability detected in the control cells. 

### 2.5. 5-Ethynyl-2’-deoxyuridine (EdU) Cell Proliferation Assay

Cells were seeded at a density of 8 × 10^3^ onto 96-well plates, and exposed to 50 μM of EdU 24 h following transfection (RiboBio, Guangzhou, China). After fixation with 4% paraformaldehyde and permeabilization with 0.5% Triton X-100, cells were incubated with 1×Apollo reaction cocktail for 30 min at room temperature followed by Hoechst 33342 staining. Cell proliferation was analyzed under an inverted fluorescent microscope (Olympus IX73, Tokyo, Japan) using cellSens Standard imaging software (Olympus, Tokyo, Japan).

### 2.6. Flow Cytometry Assay 

Based on flow cytometry assay, JC-1 (Beyotime Biotechnology, Haimen, China) staining was used to measure the mitochondrial membrane potential (Δψm) of 3T3-L1 cells according to the manufacturer’s directions. Briefly, after indicated treatments, cells were collected from 6-well plates and incubated with 1 mL JC-1 working solution for 20 min at 37 °C. Cells treated with 10 μmol/L carbonyl cyanide m-chlorophenylhydrazone (CCCP) were used as negative control. The cells were then rinsed twice with JC-1 staining buffer, and the fluorescence intensity of both mitochondrial JC-1 monomers (λ_ex_ 514 nm, λ_ex_ 529 nm) and aggregates (λ_ex_ 585 nm, λ_em_ 590 nm) were detected using flow cytometer (FC500, Beckman Coulter, Indianapolis IN, USA). The ratio of aggregated JC-1 and monomeric JC-1 represented Δψm of 3T3-L1 cells. 

### 2.7. Live Cell Fluorescent Imaging of Mitochondria

MitoTracker Red CM-H_2_X_Ros_ (Thermofisher Scientific, Waltham, MA, USA) was applied to stain mitochondria of living cells. Cells were cultured in 35 mm glass bottom culture dishes (Nest Scientific, Rahway, NJ, USA) and incubated with 200 ng/mL MitoTracker Red CM-H_2_X_Ros_ at 37 °C for 30 min. Hoechst33342 (0.5 μg/mL) was used for nucleus staining. The images were observed using a laser scanning confocal microscopy and FV10-ASW 4.2 software (Olympus FV1200, Tokyo, Japan).

### 2.8. ATP Assay/Determination of ATP Content of Cells

According to ATP Assay Kit (Beyotime, BioTECH, Haimen, China), 3T3-L1 cells were lysed using commercial lysis buffer for 5 min on ice. Following centrifugation at 12,000 × g for 5 min, reconstituted luciferase substrate was added to the supernatant and incubated for 5 min in the dark. Per 6-well plate, 50 μL of each sample was mixed with 100 μL ATP detection working dilution. The luminance (RLU) was measured using a multimode microplate reader. Standard curves were generated, and the protein concentration of each sample was determined using the Bradford Protein assay (Thermofisher Scientific, Waltham, MA, USA). Total ATP levels were expressed as nmol/mg protein. 

### 2.9. Cell Differentiation Assay and Oil Red O staining 

3T3-L1 cells were transfected with siRNA and *Fto* recombinant plasmid or with control siRNA and vector plasmid for 24 h. The cells were incubated in differentiation media I (Dulbecco’s modified eagle’s media with 10% fetal bovine serum, containing 0.25 μg/ml insulin, 1 μM Dexamethasone, and 0.5 mM 3-Isobutyl-1-methylxanthine [IBMX] (Sigma-Aldrich, St. Louis, MO, USA)) for 2 days and then cultured in differentiation media II ((Dulbecco’s modified eagle’s media containing 10% fetal bovine serum and 0.25 μg/mL insulin). The cells were fed every other day with differentiation media II until the lipid droplets were formed. Cells were fixed for 20 min in 10% formaldehyde and washed twice in phosphate-buffered saline and then stained in Oil Red O working solution (Abcam, Cambridge, MA, USA) for 1 h. The red lipid droplets were observed under the microscope and were photographed after rinsing cells twice with PBS. 

### 2.10. Statistical Analysis 

All data were presented as mean ± standard deviation, and all experiments were performed in triplicate. The statistical significance of differences was evaluated with the student’s t-test or one-way analysis of variance (ANOVA) using SPSS Statistics (Version 19.0, IBM, Armonk, NY, USA). *p* < 0.05 was considered significant.

## 3. Results 

### 3.1. Fto Gene Affects the Proliferation of 3T3-L1 Cells 

**Figure 1:**
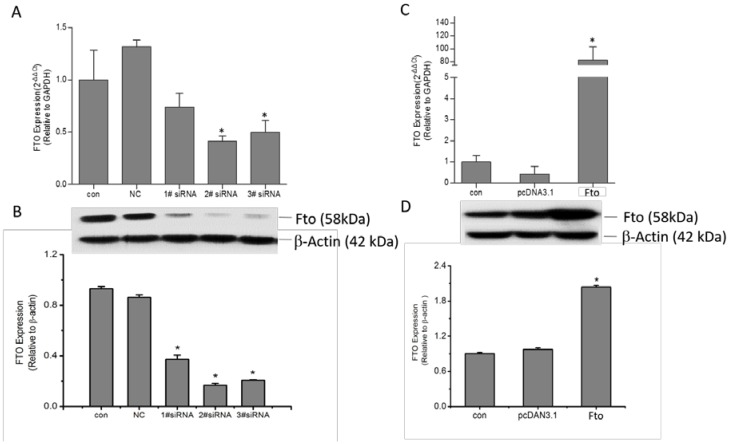
Construction of Fto knockdown or overexpressed pre-adipocyte model: (**A**) 3T3-L1 cells were transfected with siRNA control or three Fto specific siRNAs for 24 h. *Fto* mRNA was identified using qRT-PCR, with *GAPDH* as control gene. The value of 2^−∆∆CT^ was represented as Means ± SD of three independent analyses. * *p* < 0.05, *versus* NC group. (**B**) Western blot was applied to determine the Fto protein expression. The expression level was quantified as fold changes in grey value, with β-actin as the loading control. * *p* < 0.05, *versus* NC group. (**C**) The *Fto* mRNA expression and (**D**) protein expression level in 3T3-L1 cells were separately detected 24 h after transient transfection of pcDNA3.1-Fto or empty pcDNA3.1 vector, as described above. * *p* < 0.05 *versus* empty pcDNA3.1 vector group.

The expression level of mouse *Fto* was confirmed by real time PCR and Western blot assay. Our results demonstrate that Fto siRNA transfection effectively suppressed *Fto* at both mRNA and protein level (Figure 1A,B), while the recombinant plasmid transfection increased Fto expression in 3T3-L1 cells (Figure 1C,D).

**Figure 2:**
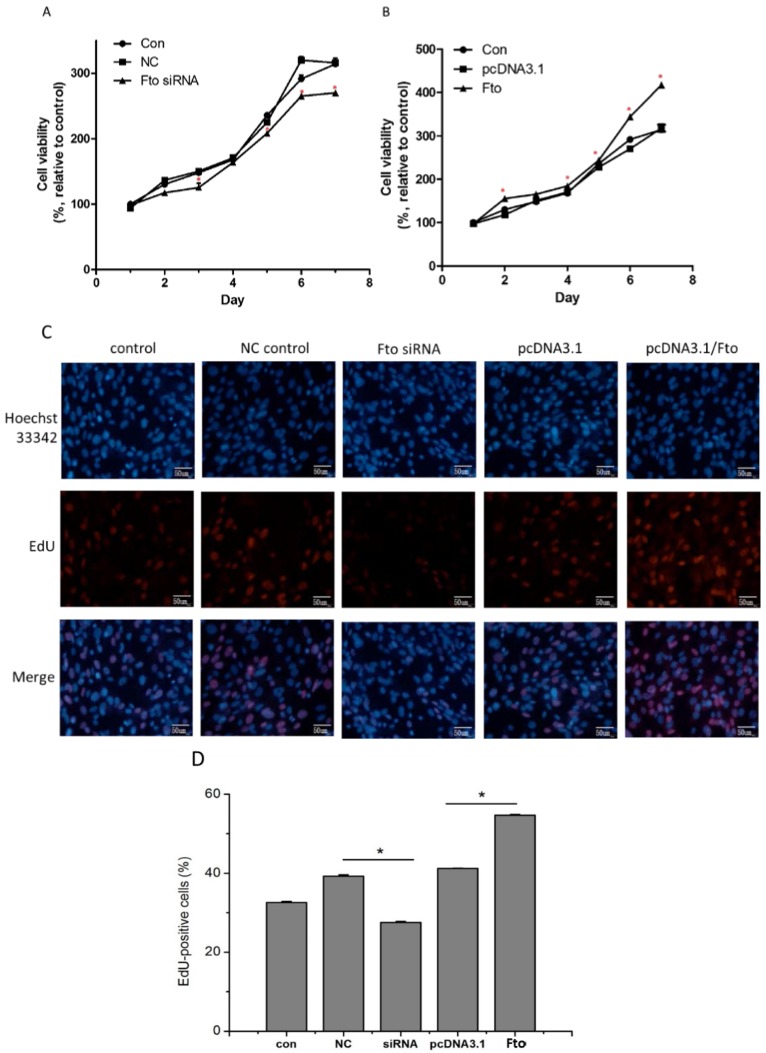
*Fto* affected 3T3-L1 cell proliferation. 3T3-L1 cells were transfected with Fto siRNA and NC siRNA (**A**), or transfected with *Fto* expression plasmid and vacant plasmid pcDNA3.1 (**B**) for 24 h. Cells were seeded in 24-well plates and were counted at Day 1 to Day 7, respectively. Cell viability was expressed as the percentage of viable cells relative to cell viability detected in the mock transfected control cells. All samples were detected in triplicate. * *p* < 0.05, *versus* control. (**C**) 3T3-L1 cells were transfected with siRNA and plasmid for 24 h, and after adipogenic induction for another 24 h, then the cell proliferation was analyzed via EdU incorporation. The representative images were shown as above; EdU (Red), Hoechest 33342 (blue); scale bar, 50 μM. (**D**) Quantification analysis of EdU positive cells.

The effect of Fto expression on the proliferation of pre-adipose cells was evaluated by MTT cell viability assay and using an EdU proliferation assay. As shown in Figure 2, when Fto expression was suppressed, the viability of 3T3-L1 cells was significantly reduced as compared to NC siRNA transfected cells (Figure 2A) (*p* < 0.05). By contrast, Fto recombinant plasmid increased the cell viability relative to the control transfected group (*p* < 0.05) (Figure 2B).

Moreover, cell EdU proliferation assay was performed 24 h after adipogenic induction. Our results indicate that pre-adipocytes with Fto-inhibition were less proliferative 24 h following adipogenesis induction as compared to NC control. However, *Fto* overexpression, induced by recombinant plasmid transient transfection, increased the proliferation of 3T3-L1 cells, as early as 24 h after adipogenesis induction (Figure 2C,D). Our data were consistent with the study by Merkestein *et al.* [20], which demonstrated that FTO acts early in adipogenesis to enhance adipocyte number. Taken together, these results demonstrate that *Fto* may regulate the proliferation of pre-adipocytes as well as the cell proliferation during early adipogenesis *in vitro*. 

### 3.2. Fto Gene Influences the Mitochondrial Membrane Potential and ATP Levels of Pre-adipocytes 

**Figure 3:**
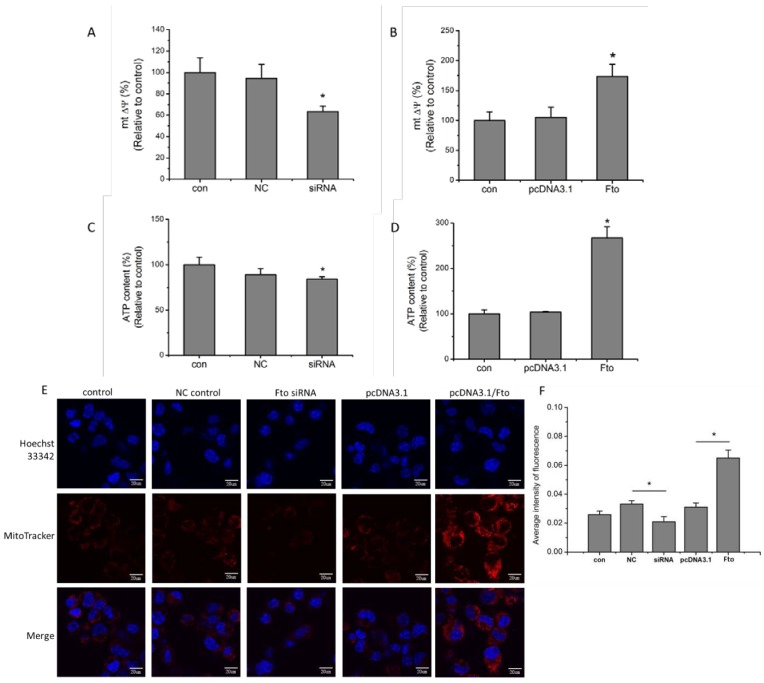
Fto influenced the mitochondrial membrane potential and ATP generation of pre-adipocytes. (**A**,**B**) Mitochondrial membrane potential of 3T3-L1 cells were determined using JC-1, 24 h after transfection. The ratio of aggregated JC-1 and monomeric JC-1 represented Δψm of cells. Results are expressed as the means ± SD from three independent experiments. * *p* < 0.05 *versus* NC/empty pcDNA3.1 vector group. (**C**,**D**) Cellular ATP concentrations were detected in 3T3-L1 cells 24 h after transfection. Results are expressed as the means ± SD from 3 independent experiments. * *p* < 0.05 *versus* NC/empty pcDNA3.1 vector group. (**E**) Parental and mock transfected control 3T3-L1 cells, together with *Fto*-deficient or *Fto*-overexpressed pre-adipocytes were incubated with MitoTracker Red CM-H_2_X_Ros_ staining, 24 h after transfection. The images were visualized by fluorescence microscopy as shown above; scale bar, 20 μm. (**F**) Quantification analysis of MitoTracker fluorescence intensity.

The cellular metabolism, mitochondrial membrane potential (MMP) and cellular ATP levels of 3T3-L1 cells were determined as previously described [23]. As shown in Figure 3, 24 h following transfection, 3T3-L1 cells with different *Fto* gene expression levels exhibited different MMP levels. Specifically, 3T3-L1 cells with suppressed *Fto* expression demonstrated a ~30% lower MMP level than NC siRNA transfected cells (Figure 3A) while MMP levels in *Fto*-overexpressed cells were 40% higher than in vector transfected control cells (Figure 3B). These results indicate that Fto is involved in the regulation on MMPs of adipocyte precursors.

Live cell imaging of mitochondria using Mitotracker Red CM-H_2_X_Ros_ staining was performed to investigate the effects of modulating Fto expression. The fluorescence dye MitoTracker Red CM-H_2_X_Ros_ is cell-permeable and sequesters in the mitochondria. This dye does not emit fluorescence unless it is oxidized; thus the observed fluorescence implies the presence of actively respiring mitochondria [24]. In the present study, decreased fluorescence was observed in *Fto*-deficient 3T3-L1 cells when compared with NC controls (Figure 3E) while enhanced MitoTracker Red fluorescence was observed in the context of *Fto*-overexpression. Specifically, *Fto*-overexpression resulted in a two-fold increase in the fluorescence intensity as compared to vector-transfected 3T3-L1 cells (Figure 3E,F).

Cellular ATP content was also evaluated. As shown in Figure 3C,D, the cellular ATP level decreased in the Fto knockdown 3T3-L1 cells while *Fto*-overexpression enhanced luminance by up to two fold compared with mock transfection groups. The results were in agreement with MMP and Mitotracker staining results and together suggested that Fto might play a role in the regulation of mitochondrial activity of pre-adipocytes. 

### 3.3. Fto Gene Regulated the Differentiation of Pre-adipocyte 

**Figure 4:**
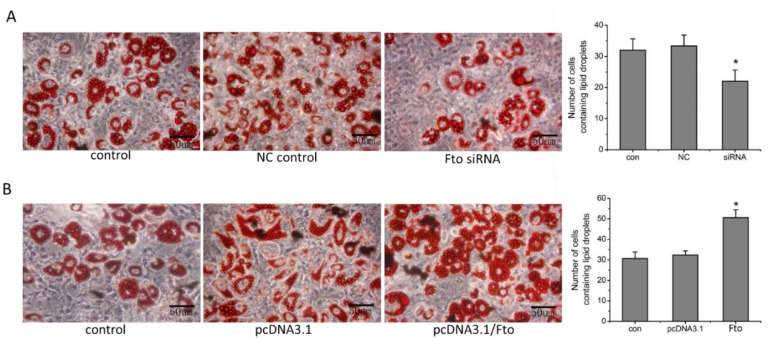
Effects of Fto on 3T3-L1 cells differentiation: (**A**) The NC control and Fto siRNA transfected 3T3-L1 cells, together with parental pre-adipocytes were treated with differentiation media at 24 h after transfection, and were subsequently incubated for 10 to 14 days. Cells were fixed and stained with Oil Red O. Then the cells were photographed under a phase contrast microscope, and five randomly selected microscopic fields for each group were then counted; scale bar, 50 μm. * *p* < 0.05 *versus* NC group. (**B**) The differentiation of *Fto* overexpressed 3T3-L1 cells were assayed as described above. * *p* < 0.05 *versus* empty pcDNA3.1 vector group.

Derived from mouse 3T3 cells, 3T3-L1 has the ability to differentiate into adipocytes. The effect of *Fto* on 3T3-L1 cells differentiation was detected using intracellular lipid droplets staining assay. As shown in Figure 4, following induction of differentiation, the lipid droplets in 3T3-L1 cells with *Fto*-suppression were irregular and smaller, and the amount of differentiated cells was significantly less than the amount observed in NC control group (*p* < 0.05). By contrast, *Fto*-overexpression led to more cellular lipid droplets and more differentiated cells as compared to the mock transfection group under typical culture conditions (*p* < 0.05). 

### 3.4. Fto Regulated the Differentiation and Proliferation of 3T3-L1 Cells via Multiple Mechanisms 

**Figure 5:**
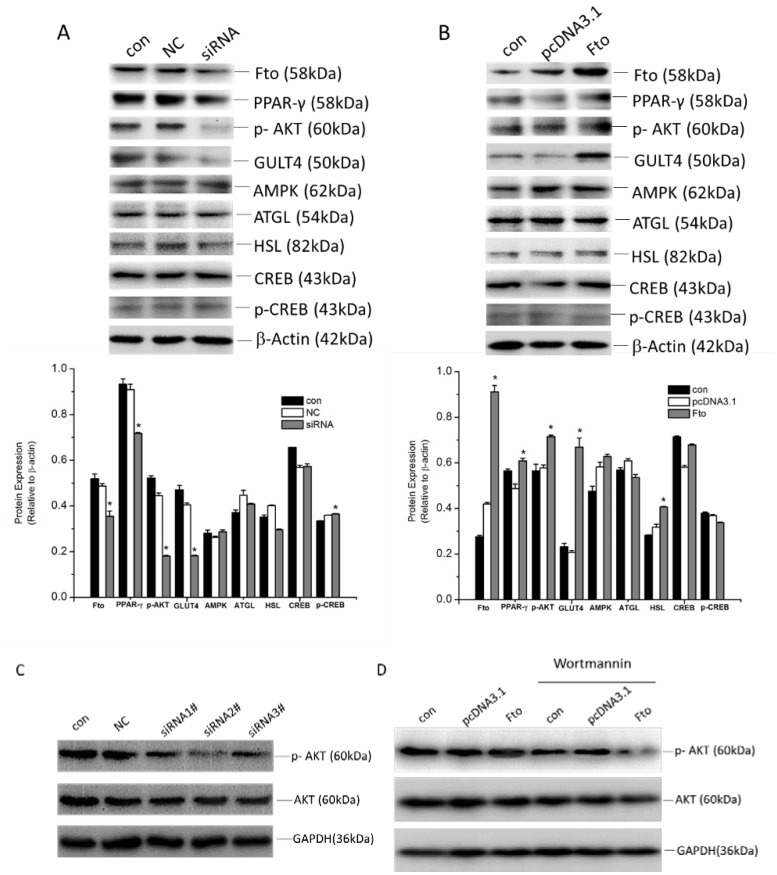
Effect of Fto on proliferation and differentiation of pre-adipocyte. (**A**) 3T3-L1 cells transfected with/without NC siRNA or Fto siRNA, as well as (**B**) *Fto* recombinant plasmid or vector “mock” transfected 3T3-L1 cells were harvested 24 h after transfection for protein extraction and quantified. Fifty microgram of total protein were analyzed by Western blot. Expressions and activities of specific transcription factors and signaling factors were detected. The expression level was displayed as fold changes in band density. Results are expressed as the means ± SD from three independent experiments. * *p* < 0.05, *vs*. the mock transfection group. (**C**) Further, three different Fto siRNA were transfected into 3T3-L1 cells for 24 h. The phosphor-Akt and Akt expression were examined by Western blot assay. (**D**) 3T3-L1 cells were transfected with vector plasmid and *Fto* plasmid for 24 h, then were cultured in the absence or presence of the PI3k inhibitor (Wortmannin, 100 nmol/L) for 24 h. The expression level of phospho-Akt and Akt was determined by Western blot analysis.

To examine the contributions of Fto in regulating differentiation and proliferation, the expression of several lipid metabolism-related transcription factors and signaling pathways were detected in *Fto* knockdown or overexpressed 3T3-L1 cells. The results showed that knockdown of Fto decreased the expression of peroxisome proliferator-activated receptor γ (PPARγ) in addition to significantly suppressing Akt phosphorylation and the expression of Glucose transporter type 4 (GLUT4) (Figure 5A,C). No obvious effects on the expression of AMP-activated protein kinase (AMPK), adipose triglyceride lipase (ATGL), hormone-sensitive lipase (HSL) and cAMP response element-binding protein CREB were observed (Figure 5A). *Fto* overexpression has the opposing effect as Fto-knockdown (Figure 5B). We noticed increased HSL in 3T3-L1 cells with elevated Fto expression. Wortmannin (100 nmol/L, Cayman Chemical, Ann Arbor, MA, USA), which is a specific inhibitor of the phosphatidylinositol 3-kinase (PI3k)/Akt signaling cascade, was utilized to investigate the effect of Fto on the proliferation and differentiation. As shown in Figure 5D, phospho-Akt was markedly reduced in *Fto*-overexpressing 3T3-L1 cells following application of Wortmannin, suggesting that Fto mediates the phosphorylation of Akt at serine 473. These results indicate that Fto might be a multi-functional regulator in pre-adipocytes proliferation and differentiation. 

## 4. Discussion

White adipose tissues are distributed throughout the whole body, and the excessive accumulation of WAT is thought to account for obesity [2,8,9]. Pre-adipocytes are the precursors of the adipocytes, which consist of WAT, and regulation of the balance between proliferation and differentiation of these cells will determine the quantity and size of mature adipocytes [12]. As such, dysregulation of proliferation and differentiation in pre-adipocytes contributes to the onset of obesity. 

Fisher *et al.* [15] previously demonstrated that mice lacking the *Fto* gene suffered from severe growth retardation. In the present study, we observed a significant inhibition of proliferation in *Fto* knockdown 3T3-L1 cells (Figure 2A,C), which is consistent with the *in vivo* results of Fisher *et al.* A newly published study by Jeffery *et al.* [25] indicated that increased proliferation of adipocyte precursors in visceral WAT during the early stage of high fat diet caused adipogenesis. Using mouse embryonic fibroblasts (MEF) derived from *Fto-*deleted or *Fto*-overexpressed mice, Merkestein *et al.* [20] found that FTO could enhance MEF proliferation as early as 48 h after adipogenic induction. In our study, by applying Fto siRNA or recombinant plasmid transfection, we demonstrated that Fto could regulate the proliferation of 3T3-L1 cell line in the precursor status, and even earlier during adipogenesis (24 h after adipogenic induction) (Figure 2A–C). These results confirmed the regulatory role of Fto in adipogenesis *in vitro*.

As a well-characterized cell model, fibroblast-like 3T3-L1 cells will differentiate into a signet ring appearance of adipocyte-like phenotype under appropriate stimulation, which represents the synthesis and accumulation of triglycerides [26]. Several studies have reported that *Fto-*overexpression can significantly increase the triglyceride content of the cell [17,27,28]. Merkestein *et al.* [20] recently reported that primary adipocytes and MEFs derived from *Fto* overexpression mice demonstrated increased potential for adipogenic differentiation while *Fto* knockout mice derivative MEFs exhibited reduced adipogenesis. In our study, following differentiation stimulation, we found *Fto*-overexpressing cells can differentiate into more mature adipocytes with more lipid droplets than vacant plasmid transfected cells (Figure 4B). By contrast, less, irregular and smaller lipid droplets were observed after differentiation induction in Fto siRNA transfected pre-adipocytes (Figure 4A). Our *in vitro* data were in agreement with the finding of Merkestein *et al.* [20] that FTO affects fat mass via regulating adipocyte differentiation *in vivo*. 

Several studies have suggested that *Fto* expression level was strongly correlated with energy status. For example, overexpression of *Fto* led to fatty acid accumulation and increased food intake and thus resulted in obesity [17]. Tews *et al.* [19] recently reported that FTO is involved in WAT browning in human SGBS pre-adipocytes. Although they showed no difference in the rate of differentiation and triglyceride content between control and FTO-deficient SGBS cells, they determined that FTO deficiency could lead to mitochondrial uncoupling and increase energy expenditure [19]. In the present study, we found that Fto could regulate the MMP of mouse pre-adipocyte 3T3-L1 and affect ATP generation in 3T3-L1 cells. Our MTT assay results also indicated effects of Fto on the mitochondrial activity of live 3T3-L1 cells, since the conversion of MTT into formazan requires the functioning mitochondria. Based on these results, we hypothesize that FTO might affect the mitochondrial function *in vitro*. However, considering that Tews *et al.* did not show significantly varied citrate synthase activity, mitochondrial contents and structure in FTO deficient SGBS cells [20], we should carefully analyze the effects of Fto on mitochondrial function in our model system in future studies to fully characterize the implications of our findings. Nevertheless, our results clearly indicated that Fto could represent a functional regulator on the 3T3-L1 pre-adipocyte in the context of obesity.

There are numerous opinions regarding the formation of adipose tissue in the development and expansion of adipose tissue in obesity [25]. Jeffery *et al.* [25] recently suggested that the formation of adipocytes in obesity and development is controlled by distinct molecular mechanisms. Fat accumulation and morphology changes of adipocytes were thought to represent a consequence of specific genes expression, including several transcription factors, positive and negative signaling pathways effectors, and even non-coding RNAs, which were induced during the process of pre-adipocytes proliferation and differentiation [11,12]. In this study, we wanted to identify molecular mechanisms and signaling pathways that regulated pre-adipocyte proliferation and differentiation via Fto. 

Our data demonstrate that *Fto* overexpression induced the expression of transcription activator PPARγ, while Fto deficiency impaired PPARγ expression in 3T3-L1 pre-adipocyte. PPARγ belongs to the ligand-activated nuclear receptor superfamily, which is preferentially expressed in adipocytes [29], and is vital in expression regulation of the gene networks involved in adipogenesis, lipid metabolism, inflammation and maintenance of metabolic homeostasis [29,30]. In 2013, Bravard *et al.* [31] hypothesized that FTO might participate in the increased expression of PPARγ2 during adipogenesis via demethylating its promoter region, but the subsequent study failed to verify their hypothesis. Further studies are required to clarify the mechanistic actions of FTO on PPARγ expression regulation during adipogenesis. 

We found Fto may also be involved in Akt activity regulation in 3T3-L1 cells. The insulin-mediated activation of PI3K/Akt signaling pathway plays a crucial role in energy metabolism [32,33,34]. Activated Akt can induce glycogen synthesis by inhibiting GSK-3, promote protein synthesis via mTOR cascades, and facilitate cell survival through inhibiting pro-apoptotic factors [32,34]. The insulin receptor signaling is also important for fatty acid and cholesterol synthesis [35]. The activation of adipocyte precursors is thought to dependent on the PI3K-AKT2 pathway in multiple models of obesity, but Jeffery *et al.* [25] demonstrated that WAT-development does not require AKT2. However, using a specific PI3k inhibitor, we found that Akt signaling is involved in Fto regulated adipogenesis *in vitro* (Figure 5). Moreover, in the present study, the regulation of Fto on Akt activity could in part interpret the variations of proliferation, energy homeostasis, and adipogenesis in 3T3-L1 cells with different Fto levels. Besides, Fto expression levels similarly affected GLUT4 expression, which also participates in insulin receptor signaling [36]. We were also able to conclude that FTO had a minor effect on HSL expression (following *Fto*-overexpression) and no effect on the expression of AMPK and ATGL. 

In summary, our study demonstrates that Fto can affect the proliferation, energy homeostasis, and differentiation of 3T3-L1 pre-adipocyte. Fto might function as a regulator of PI3K Akt signaling and PPARγ expression. While our results have improved our understanding of the importance of Fto on obesity, several questions remain to be answered. Specifically, further studies are required to determine if the Fto-mediated effect on PPARγ is mediated through demethylation activity or via other mechanism. Similarly, the exact mechanisms through which Fto affects Akt phosphorylation requires attention. More detailed investigations using more sophisticated model system (primary adipocytes, embryonic fibroblasts, *etc.*) are extremely necessary to uncover the molecular mechanisms underlying Fto’s role in adipogenesis and obesity.
